# Bio-Humoral and Non-Invasive Haemodynamic Correlates of Renal Venous Flow Patterns across the Heart Failure Spectrum

**DOI:** 10.3390/medicina59101704

**Published:** 2023-09-24

**Authors:** Lavinia Del Punta, Nicolò De Biase, Matteo Mazzola, Francesco Filidei, Alessio Balletti, Silvia Armenia, Valerio Di Fiore, Simona Buralli, Gian Giacomo Galeotti, Marco De Carlo, Cristina Giannini, Stefano Masi, Nicola Riccardo Pugliese

**Affiliations:** 1 Department of Clinical and Experimental Medicine, University of Pisa, 56124 Pisa, Italy; 2 Department of Pathology, Cardiology Division, University of Pisa, Via Paradisa 2, 56124 Pisa, Italy; 3 Cardiac, Thoracic and Vascular Department, Azienda Ospedaliero-Universitaria Pisana, 56126 Pisa, Italy; marcodecarlo@gmail.com (M.D.C.);

**Keywords:** heart failure, renal venous flow, congestion

## Abstract

*Background*: We evaluated the bio-humoral and non-invasive haemodynamic correlates of renal congestion evaluated by Doppler renal venous flow (RVF) across the heart failure (HF) spectrum, from asymptomatic subjects with cardiovascular risk factors (Stage A) and structural heart disease (Stage B) to patients with clinically overt HF (Stage C). *Methods*: Ultrasound evaluation, including echocardiography, lung ultrasound and RVF, along with blood and urine sampling, was performed in 304 patients. *Results*: Continuous RVF was observed in 230 patients (76%), while discontinuous RVF (dRVF) was observed in 74 (24%): 39 patients had pulsatile RVF, 18 had biphasic RVF and 17 had monophasic RVF. Stage C HF was significantly more common among patients with dRVF. Monophasic RVF was associated with worse renal function and a higher urinary albumin-to-creatinine ratio (uACR). After adjusting for hypertension, diabetes mellitus, the presence of Stage C HF and serum creatinine levels, worsening RVF patterns were associated with higher NT-proBNP levels, worse right ventricular–arterial coupling, larger inferior vena cava and higher echo-derived pulmonary artery wedge pressure. This trend was confirmed when only patients with HF Stage C were analysed after adjusting for the left ventricle ejection fraction (LVEF). *Conclusion*: Abnormal RVF is common across the HF spectrum. Worsening RVF patterns are independently associated with increased congestion, worse non-invasive haemodynamics and impaired RV-arterial coupling. RVF evaluation could refine prognostic stratification across the HF spectrum, irrespective of LVEF.

## 1. Introduction

Heart failure (HF) is a complex syndrome with cardinal symptoms and signs that result from structural and functional abnormalities of the heart, leading to elevated intracardiac pressures and/or inadequate cardiac output [1,2]. Increased pulmonary capillary pressure (>15 mmHg), conventionally measured by pulmonary arterial wedge pressure (PAWP), is recommended by international guidelines as the diagnostic criterion for left-sided HF [1,2]. However, the interaction between the left and right ventricles determines, in turn, a strong relationship between PAWP and central venous pressure (CVP) [3]. Backwards filling pressure transmission towards the pulmonary vascular bed and peripheral venous system (PAWP and CVP, respectively) is responsible for fluid accumulation in the intravascular compartment and interstitial space, representing the haemodynamic phase of end-organ congestion [4,5]. Renal congestion caused by increased CVP represents one of the main pathophysiologic mechanisms in HF, leading to raised renal interstitial pressures, partial collapse of nephrons, ischaemia and neurohormonal activation [6,7]. Renal venous flow (RVF) is normally continuous with a small varying amplitude during the cardiac cycle [5,8]. Recently, Doppler ultrasound at the level of interlobular veins has exhibited high clinical feasibility and acceptable reproducibility [5,8,9]. With increasing CVP in HF patients, venous compliance decreases secondary to intravascular volume expansion and the increase in renal interstitial pressure within the encapsulated kidneys. These haemodynamic alterations progressively affect RVF during the cardiac cycle, leading to a discontinuous pattern [5,8,9,10]. In patients with HF, regardless of left ventricle ejection fraction (LVEF), RVF derangements evaluated with Doppler echocardiography are strongly associated with higher CVP [9]. The development of a pathological RVF pattern also seems to represent an earlier marker of congestion compared to the standard echocardiographic surrogates of intracardiac pressures [10]. Moreover, renal venous congestion impairs glomerular and tubular physiology due to severe metabolic and haemodynamic changes within the renal parenchyma, including glomerular hypertension, tissue hypoxia and neuro-humoral derangements [6,11]. We recently demonstrated in outpatients with HF that the assessment of multi-organ congestion, including RVF, can improve risk stratification [5].

In this study, we evaluated the clinical, bio-humoral and non-invasive haemodynamic correlates of all the different RVF patterns across the HF spectrum from asymptomatic subjects with cardiovascular risk factors (Stage A) and structural heart disease (Stage B) to patients with clinically overt HF (Stage C) [2]. We hypothesized that RVF assessment might serve as a novel marker to identify patients with a high-risk profile regardless of LVEF and established HF diagnosis.

## 2. Methods

**Figure 1:**
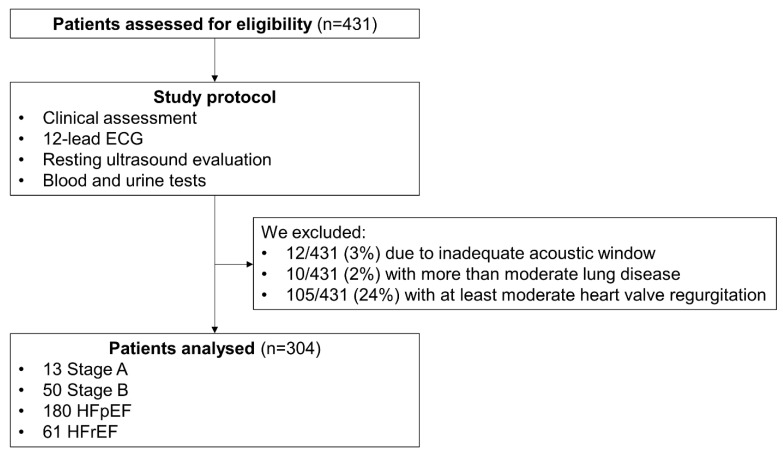
The enrolment flowchart. HFpEF: heart failure with preserved ejection fraction. HFrEF: heart failure with reduced ejection fraction.

**Study population.** We prospectively enrolled 431 consecutive outpatients referred for dyspnoea to the University Hospital of Pisa between March 2021 and December 2022. All patients underwent a complete clinical assessment, blood and urine tests, a 12-lead ECG and an ultrasound evaluation. We excluded 12 patients (3%) with inadequate acoustic windows (i.e., poor-quality images). All the patients with a history of lung disease underwent spirometry, and those (*n* = 10, 2%) with more than moderate airflow obstruction were excluded. Patients with at least moderate heart valve regurgitation (*n* = 105, 24%) were also excluded. All patients were stable at the time of recruitment. The final study population (*n* = 304) consisted of 13 subjects in Stage A, 54 in Stage B and 241 patients with Stage C HF, who were stratified into HF with reduced (LVEF <50%, HFrEF, *n* = 61) and preserved LVEF (≥50%, HFpEF, *n* = 180; Figure 1).

The study fulfilled the requirements in the Declaration of Helsinki; the protocol was approved by the local ethics committees (number 19204), and written informed consent was obtained from all patients.

Laboratory evaluation. Patients were instructed to fast overnight and to not take any medications before blood and urine sampling on the morning of the tests. Blood samples were drawn after a 30 min supine rest. Detailed laboratory protocol is provided in the Supplementary Materials. After blood sampling, patients were asked to give a urine sample. We estimated the fractional excretion of sodium (FENa) as [12]:

$$\frac{\mathrm{urine}\;\mathrm{sodium}\;\times \;\mathrm{serum}\;\mathrm{creatinine}}{\mathrm{serum}\;\mathrm{sodium}\;\times \;\mathrm{urine}\;\mathrm{creatinine}}\%$$

We defined micro-albuminuria and macro-albuminuria as a urinary albumin-to-creatinine ratio (uACR) >30 mg/g and >300 mg/g, respectively. We evaluated the instantaneous estimated plasma volume status (ePVS) in mL/g [13] as:

$$\frac{1-hematocrit}{hemoglobin}\times 100$$

**Baseline echocardiography.** All patients underwent a comprehensive transthoracic echocardiography examination with a phased array transducer (1–5 MHz, Hitachi Medical Systems LISENDO 880, Tokyo, Japan) according to the international recommendations, including 3D and speckle tracking evaluation (STE) [14]. We non-invasively estimated echo-derived pulmonary artery wedge pressure (ePAWP) and pulmonary vascular resistance using a previously validated equation [15]. A detailed echocardiographic protocol is provided in the Supplementary Materials.

**Figure 2:**
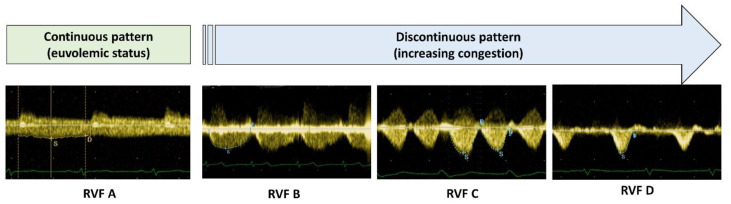
Assessment of renal venous flow (RVF) using Doppler ultrasound. The transition from a continuous RVF (pattern A) to discontinuous (pattern B to D) is an expression of worsening renal congestion.

**Renal venous flow (RVF).** Doppler assessment of RVF was performed in the left lateral decubitus position, using the same phased array transducer aligned with the lowest intercostal space to obtain a longitudinal view of the right kidney. The flow scale of colour Doppler imaging was adjusted to low-flow velocities (<20 cm/s) to optimize the identification of the interlobar veins. The best-aligned vein was then sampled with pulsed-wave Doppler during an end-expiratory breath hold. The scale was adjusted to maximize the signal amplitude (usually around ≈ 20 cm/s), and the electrocardiographic signal was used to synchronize the RVF signal with the cardiac cycle. We used a semi-quantitative assessment of the effects of central venous pressure (CVP) on renal haemodynamics. In normal conditions, the interlobar RVF is continuous (cRVF; i.e., pattern A) with a small varying amplitude during the cardiac cycle (Figure 2).

With worsening congestion, the amplitude variation increases with the minimal velocity gradually approaching zero, eventually leading to a discontinuous RVF (dRVF), which could be described as pulsatile (pattern B), biphasic (pattern C) or, in the most severe cases, monophasic during diastole (pattern D) [8]. We also measured the renal venous impedance index (VII) and the venous discontinuity index (VDI). The VII is the ratio of the difference between the maximum and minimum velocity to the maximum velocity during a cardiac cycle with a number varying from 0 (no variation in velocity) to 1 (minimum velocity is 0, i.e., the flow becomes discontinuous). VDI expresses the percentage of no-flow time during a cardiac cycle [8].

**Lung ultrasound (LUS).** B-lines were measured with a linear transducer in parallel orientation (transverse) to the ribs at an imaging depth of ∼15–18 cm using an eight-region scan [8]. In each region, B-lines were counted one by one if distinguishable; if confluent, we estimated their number by the percentage of space occupied on the screen divided by 10 (up to a max of 10 B-lines/region). The sum of B-lines from the eight scanning regions yielded a score denoting the extent of the extravascular lung water; zero was defined as a complete absence of B-lines.

**Statistical analysis.** Categorical data are presented as percentages and were compared using Pearson’s chi-square test or Fisher’s exact test. Continuous data are reported as the mean ± standard deviation or median and interquartile range (IQR) for normally or skewed distributed variables, respectively. Continuous variables from two data sets were compared using Student’s *t*-test or the Mann–Whitney U test for non-normal distributions. An ANOVA test or the Kruskal–Wallis test was used to test the differential distribution of data among groups and post hoc tests were performed with Bonferroni corrections (using *p*-values at the significance levels of <0.01 and <0.05 for the ANOVA or Kruskal–Wallis tests). ANCOVA was used to adjust ANOVA results for significant covariates. Missing data were not included in the models. All tests were two-sided, with a *p*-value of <0.05 considered significant. Data were analysed with SPSS version 25.0 (IBM Corp., Armonk, NY, USA) and R 3.6.2 (R Foundation for Statistical Computing, Vienna, Austria).

## 3. Results

**Study population.** A cRVF was assessed in 230 patients (76%), while dRVF was assessed in 74 (24%), identifying pattern B in 39 patients, pattern C in 18 patients and pattern D in 17 patients. The demographic and clinical characteristics of the whole population and according to the presence of cRVF versus dRVF are presented in Table 1. 

Overt HF was significantly more common among patients with dRVF than cRVF; only four patients in Stages A/B showed a dRVF (all with pattern B). The dRVF cohort more often displayed clinical signs of congestion and reported a significantly worse quality of life as evaluated by the KCCQ score. Atrial fibrillation (AF) was significantly more common in those with dRVF, as well as using mineralocorticoid receptor antagonists and loop diuretics.

**Laboratory evaluation.** In patients with dRVF, creatinine serum levels were significantly higher, and eGFR was significantly lower than those with cRVF (Table 1). NT-proBNP levels were significantly higher in the dRVF cohort, even after excluding patients with AF, as were uric acid and high-sensitivity C-reactive protein levels. The ePVS, aldosterone and high-sensitivity Troponin T levels were also significantly higher in patients with dRVF. Finally, spot urinary sodium and FENa were significantly lower in the dRVF group. There were no significant differences in the prevalence of micro- or macro-albuminuria between the two subgroups.

**Ultrasound evaluation.** While LV volumes and masses were similar between the two subgroups, LVEF and LV global longitudinal strain were impaired in patients with dRVF (Table 2). 

The same patients displayed worse LV diastolic function (i.e., higher mitral E-wave and E/e’ ratio), greater LA volume, lower LA reservoir strain, higher pulmonary pressures and pulmonary resistances, as well as worse right ventricle (RV) systolic function and RV-pulmonary arterial coupling (i.e., lower tricuspid annular plane systolic excursion [TAPSE]/systolic pulmonary artery pressure [sPAP]). Furthermore, IVC dilation was more common, and the B-lines count was higher in patients with dRVF than in patients with cRVF. The median VII was 0.26 (0.21–0.35) in patients with cRVF, while the median VDI was 14.3% (6.8–39%) in those with dRVF, ranging from 8.6% (5.1–14.3%) in RVF pattern B to 19.6% (11.6–25.1%) in RVF pattern C and 57% (42–69.6%) in RVF pattern D (*p* < 0.0001).

Among patients with HF Stage C, dRVF was associated with the worst laboratory and ultrasound indices of congestion. In contrast, patients with HF Stage C and cRVF had intermediate characteristics between HF Stage C with dRVF and Stages A/B (Supplemental Table S1).

**Figure 3:**
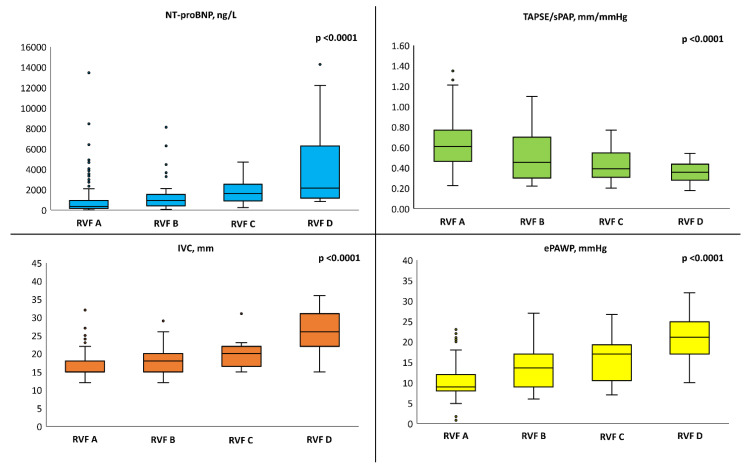
Box-and-whisker plots showing distributions of NT-proBNP, TAPSE/sPAP, IVC diameter and ePAWP in the whole population according to different renal venous flow (RVF) patterns. Analyses are adjusted for hypertension, diabetes mellitus, a definite diagnosis of heart failure (i.e., HF Stage C) and serum creatinine. ePAWP: echo-derived pulmonary artery wedge pressure; NT-proBNP: N-terminal pro-brain natriuretic peptide; sPAP: systolic pulmonary artery pressure; TAPSE: tricuspid annular plane systolic excursion.

**Analysis of individual RVF patterns.** After adjusting for hypertension, diabetes mellitus, a definite diagnosis of HF (i.e., HF Stage C) and serum creatinine, worsening RVF patterns were associated with higher NT-proBNP serum levels, lower TAPSE/sPAP, larger IVC diameter and higher ePAWP (Figure 3).

In particular, RVF pattern D consistently showed the worst features, including significantly worse renal function and higher uACR. RVF patterns B and C displayed similar characteristics, which resulted in an intermediate phenotype between RVF A and RVF D (Table 3). 

This trend was confirmed in a sensitivity analysis including only patients with HF Stage C after adjusting for LVEF (Supplemental Table S2). RVF pattern D was also associated with worse congestion indexes among the subgroup of patients with AF (Supplemental Table S3).

## 4. Discussion

This is the first study to investigate clinical, bio-humoral and echocardiographic correlates of different RVF patterns across the HF spectrum, from Stage A to Stage C. The assessment of RVF is reliable and time-saving (as it requires <5 min) and provides a semi-quantitative grading [5,8]. We observed that dRVF is associated with a significantly poorer reported quality of life, worse biventricular systo-diastolic function, worse renal function and higher levels of NT-proBNP, regardless of LVEF. The distinction among different RVF patterns reflects not only a more compromised renal haemodynamic but also a more advanced heart disease, according to multiple laboratory and ultrasound indices of congestion and cardiovascular dysfunction. In particular, RVF pattern D is associated with the worst clinical, bio-humoral and haemodynamic phenotype. The prevalence of AF is significantly higher in patients with dRVF and may act as a confounder. However, a sensitivity analysis confirmed the ability of RVF patterns to stratify patients with AF.

**RVF and haemodynamics.** The dRVF pattern was more prevalent in patients with established HF diagnosis, regardless of LVEF, resulting from the backwards transmission of increased CVP [3,8,9,10]. Although increased PAWP represents the main haemodynamic characteristic of left-sided HF, under normal conditions CVP and PAWP are strictly related; indeed, there is a 1.5 mm Hg increase in PAWP for every 1 mm Hg increase in CVP during exercise [16,17]. Coping with an elevated pulmonary pressure regimen to maintain stroke volume, RV primarily engages homeometric and heterometric compensatory mechanisms, which increase contractility [18]. However, in the presence of LV disease, the loss of positive systolic interaction with the LV and/or the extension of the disease process to RV myocardial tissue prevent a proper adaptation, leading to the development of RV-arterial uncoupling [19]. Therefore, RV dilation occurs at the expense of higher levels of CVP, which eventually affects renal perfusion and RVF [8,9,10]. Iida et al. were the first to systematically examine RVF in 217 patients with HF, regardless of LVEF, either following a hospital admission or in the outpatient setting. They demonstrated that more advanced RVF patterns were associated with higher mean right atrial pressure, used as a surrogate of CVP, underpinning a solid relationship between significant tricuspid regurgitation and the monophasic pattern [9]. In the present study, we excluded patients with any significant heart valve regurgitation. Nevertheless, we observed that dRVF is associated with higher pulmonary pressures and non-invasive estimated PVR, worse RV systolic function and a higher prevalence of IVC dilation, confirming the close relationship of impaired RVF with RV haemodynamics and function. Moreover, using TAPSE/sPAP as an echocardiographic surrogate for the RV length–force relationship with its well-known prognostic value in HF and robust invasive haemodynamic validation [20,21], our results support the role of RV-arterial uncoupling and systemic venous congestion in the derangement of RVF. Indeed, in our population, patients with dRVF displayed more signs of extravascular lung water (B-lines) and diastolic dysfunction, with higher values of estimated PAWP as compared to those with cRVF. These findings might be partially explained by the complex interaction involving RV-arterial coupling, the lymphatic system and ventricular interdependence [3]. In experimental animal preparations [22,23,24] and clinical settings [25], increased CVP has been demonstrated to enhance EVLW accumulation in the context of elevated PAWP by hampering the ability of lymphatics to drain fluids from the lung interstitium to the subclavian venous system. Moreover, since CVP approximates pericardial pressure, this parameter can also be taken as an estimate of the extramural pressure applied to the left heart [26]. Indeed, as RV dilation occurs at the expense of systemic venous congestion because of RV-arterial uncoupling within the restricted pericardial space, ventricular interaction amplifies, requiring higher PAWP values to achieve a given transmural pressure [26]. Thus, dRVF in HF patients seems to characterize a severely deranged haemodynamic profile with elevated biventricular filling pressures and combined pulmonary and systemic congestion. This background promotes an inflammatory response, neuro-humoral activation and sympathetic-mediated vasoconstriction, which in turn contribute to the expansion of the intravascular volume through reduced venous capacitance and increased renal reabsorption of salt and water, leading to further deterioration of haemodynamics [3,27,28,29]. Noteworthy, this vicious circle is also supported by the observation of higher NT-proBNP levels with worsening RVF, even when considering only patients with AF.

**RVF and HF stages.** After adjusting for covariates including hypertension, diabetes mellitus, HF Stage C and serum creatinine, worsening of RVF remained associated with worse ultrasound and bio-humoral indexes of congestion, worse non-invasive haemodynamic variables and reduced RV-arterial coupling. Elevated levels of natriuretic peptides (i.e., NT-proBNP) [30], the presence of echocardiographic signs of EVLW (i.e., B-lines) [31,32,33,34], systemic venous congestion (i.e., IVC diameter) [35] and RV-arterial uncoupling (i.e., TAPSE/sPAP) [20,36,37] have well-known prognostic value in patients with definite HF diagnosis, regardless of LVEF. Accordingly, in the report by Iida et al., RVF patterns strongly correlated with clinical outcomes, independent of right atrial pressure and other risk factors, with pattern D displaying the worst prognosis [9]. Noteworthy, the previous observations focused on patients with definite HF diagnosis (stage C) and excluded early HF stages (Stages A/B). Our results show that impaired RVF characterizes high-risk patients across the whole HF spectrum. Nijst et al. evaluated the effect of volume loading and diuretics on RVF in 50 patients with stable HFrEF and HFpEF [10]. After volume loading, the number of patients with discontinuous RVF increased from 32% to 80% without any significant change in IVC-estimated CVP, perhaps suggesting that RVF may represent an earlier marker of the development of congestion, potentially useful for the detection of those patients without an established HF diagnosis, at risk for future transition to an overt clinical phase. We recently demonstrated that a simultaneous assessment of pulmonary, venous and kidney congestion by ultrasound could identify a high prevalence of sub-clinical congestion associated with poor outcomes, irrespective of LVEF [5]. Thus, RVF evaluation could be potentially helpful for detecting patients at higher risk of transition to overt HF or more advanced stages of HF. In the future, larger studies would be needed to investigate whether echographic RVF evaluation could have an incremental value when combined with renowned prognostic scores in HF [38,39,40], possibly above and beyond eGFR and albuminuria.

**RVF and renal function.** Patients with dRVF display higher serum creatinine levels and lower eGFR than those with cRVF. After adjusting for multiple confounding variables, the monophasic RVF pattern (RVF D) was associated with the worst renal function. Conversely, there was no apparent significant effect of RVF on the prevalence of macro- and micro-albuminuria. RVF is a highly dynamic parameter influenced by systemic and local haemodynamic changes, especially following diuretic therapy [10,41,42]. It is conceivable that a long-standing exposure of renal parenchyma to elevated CVP is necessary to increase pressure within the rigid renal capsule, leading to compression of intra-renal structure and progressive tissue remodelling, resulting in a significant tubular dysfunction [6,7,43]. Indeed, using a murine model of isolated abdominal venous hypertension, Cops et al. demonstrated that splanchnic congestion retrogradely conducted glomerular hypertension without substantial signs of tubular damage and fibrosis after twenty-one weeks [44]. Thus, this cross-sectional study may have failed in observing this association, which could be better analysed with longitudinal studies.

**Limitations.** This is a single-centre study from a tertiary referral centre: it has inherent flaws related to selection and referral bias and the absence of a validation cohort. Furthermore, as an observational study, causality cannot be deduced based on these data, especially since RVF evaluation using ultrasound is a relatively novel technique. For these reasons, larger prospective studies, as well as validation in an external cohort, are needed to confirm our preliminary results. We classified all patients with LVEF <50% as HFrEF because only 10 cases had mildly reduced LVEF (41–49%); given the small sample, we reckon that the strength of a subanalysis in this group would be limited. A significant number of patients initially enrolled were excluded due to the presence of at least moderate heart valve regurgitation. While we acknowledge that this could represent evaluation bias, we chose this criterion to eliminate a major confounding factor in our analysis, as in these patients, RVF abnormalities could be (at least partially) caused by the valve disease rather than reflect general haemodynamics. We used spot samples to assess urinary sodium, as 24 h urine collection was impractical; spot samples are easy to obtain and clinically valid [45]. The study could not determine how concomitant factors such as intrinsic renal pathology contributed to RVF patterns; however, we measured uACR to estimate kidney tubule injury. While the present population may partially overlap with the cohort of patients of our previous report (151/304, 49%) [5], the different study variables and end-points ensure the originality of the data.

## 5. Conclusions

Abnormal RVF is common across the HF spectrum. Worsening RVF patterns are independently associated with increased congestion, worse non-invasive haemodynamics and impaired RV-arterial coupling. Therefore, RVF assessment could be a novel tool to detect clinically asymptomatic patients with HF at risk for transition to more advanced stages of HF. Thanks to its convenience and execution speed, the assessment of RVF by ultrasound may assist physicians in gauging disease severity and trajectory, as well as in subjects without a definite clinical diagnosis of HF.

## Figures and Tables

**Table 1:** Population characteristics according to the presence of continuous *versus* discontinuous RVF.

Variable	Whole Population (*n* = 304)	Continuous RVF (*n* = 230)	Discontinuous RVF (*n* = 74)	*p*-Value
Demographics (0 missing)				
Age, years	76 (66–82)	76 (65–81)	76 (68–83)	0.35
Men	176 (58)	130 (57)	46 (62)	0.39
BMI, Kg/m^2^	26.5 ± 4.7	26.6 ± 4.6	26.4 ± 5.0	0.79
BSA, m^2^	1.9 ± 0.2	1.9 ± 0.2	1.9 ± 0.2	0.23
Smoker	53 (17)	43 (19)	10 (14)	
HF Stages				<0.0001
Stages A/B	63 (21)	59 (26)	4 (5)	
Stage C-HFpEF	180 (59)	134 (58)	46 (62)	
Stage C- HFrEF	61 (20)	37 (16)	24 (33)	
NYHA class				0.11
I	128 (42)	104 (45)	24 (32)	
II	135 (44)	97 (42)	38 (51)	
III	41 (14)	29 (13)	12 (16)	
KCCQ score	71 (53–82)	72 (55–84)	62 (47–76)	0.014
Arterial hypertension	212 (70)	167 (73)	45 (61)	0.08
Diabetes mellitus	67 (22)	49 (21)	18 (24)	0.55
CAD	70 (23)	55 (24)	15 (20)	0.55
Previous MI	40 (13)	28 (12)	12 (16)	0.35
Pacemaker	39 (13)	28 (12)	11 (15)	0.52
ICD/CRT	36 (12)	24 (10)	12 (16)	0.17
AFib	63 (21)	30 (13)	33 (44)	<0.0001
Clinical evaluation (0 missing)				
Brachial systolic BP, mmHg	130 ± 19	131 ± 20	127 ± 17	0.25
Brachial diastolic BP, mmHg	78 ± 13	77 ± 12	78 ± 13	0.90
Heart rate, beats/min	77 ± 11	77 ± 12	76 ± 10	0.56
No clinical signs of congestion	183 (67)	163 (77)	17 (23)	<0.0001
Pitting oedema (any degree)	74 (27)	49 (21)	30 (41)	0.001
Lung crackles (any degree)	18 (7)	4 (2)	16 (22)	<0.0001
Jugular vein distension (any degree)	14 (5)	1 (1)	13 (20)	<0.0001
Blood tests (0 missing)				
Haemoglobin, g/dL	12.7 ± 2.0	12.7 ± 2.0	12.7 ± 1.8	0.97
Creatinine, mg/dL	0.97 (0.81–1.25)	0.92 (0.79–1.20)	1.08 (0.89–1.36)	0.005
eGFR, mL/min/1.73 m^2^	62 (52–81)	69 (53–83)	59 (46–76)	0.038
BUN, mg/dL	20 (16–25)	20 (16–24)	24 (18–29)	0.001
ePVS, mL/g	4.9 (4.2–5.5)	4.8 (4.2–5.3)	5.2 (4.5–5.9)	0.01
Plasma osmolality, mOsm/kg	294 (291–300)	294 (291–299)	297 (293–303)	0.06
Na^+^, mEq/L	141 (139–142)	141 (139–142)	141 (139–142)	0.87
K^+^, mEq/L	4.35 (4.01–4.66)	4.33 (4.03–4.63)	4.36 (3.99–4.71)	0.73
Total cholesterol, mg/dL	165 ± 41	167 ± 41	158 ± 40	0.11
HbA1c, mmol/mol	41 ± 11	41 ± 12	41 ± 8	0.99
Uric acid, mg/dL	5.8 (4.6–6.6)	5.6 (4.4–6.4)	6.2 (4.9–7.7)	<0.0001
hs-CRP, mg/dL	0.16 (0.09–0.40)	0.15 (0.07–0.36)	0.24 (0.10–0.70)	0.045
IL-6, pg/mL	1.60 (0.01–4.20)	1.45 (0.10–3.58)	2.10 (0.50–5.90)	0.08
Norepinephrine, pg/mL	230 (155–335)	223 (160–321)	240 (117–370)	0.79
Renin, mIU/L	14. 8 (7.0–63.4)	14.6 (7.0–55.4)	22.0 (6.2–66.6)	0.61
Aldosterone, ng/dL	11.0 (8.4–16.4)	10.4 (7.6–15.4)	15.2 (9.8–20.8)	0.010
NT-proBNP, pg/mL	456 (193–1279)	332 (160–918)	1195 (517–2203)	<0.0001
NT-proBNP, pg/mL (sinus rhythm only)	316 (142–738)	291 (118–591)	548 (348–1601)	<0.0001
hs-Troponin T, pg/mL	17 (9–29)	15 (9–24)	27 (14–39)	<0.0001
Urine test (12 missing)				
Urine osmolality, mOsm/kg	562 (414–654)	542 (413–656)	602 (500–632)	0.71
uACR, mg/g	23 (8–66)	22 (7–63)	28 (11–98)	0.23
Albuminuria				0.50
Micro-albuminuria §	74 (27)	52 (25)	22 (34)	
Macro-albuminuria §	25 (9)	21 (10)	4 (6)	
Spot urinary sodium, mEq/L	81 (55–115)	88 (60–126)	70 (41–88)	<0.0001
FENa, %	0.55 (0.26–0.88)	0.56 (0.34–0.92)	0.44 (0–22–0.77)	0.001
Therapy (0 missing)				
Beta-Blocker	204 (67)	147 (64)	57 (77)	0.027
DHP CCB	59 (19)	47 (20)	12 (16)	0.44
ACEi or ARB	167 (55)	128 (56)	39 (53)	0.71
MRAs	91 (30)	55 (24)	36 (49)	<0.0001
ARNI	32 (11)	24 (10)	8 (11)	0.91
Statins	159 (52)	124 (54)	35 (47)	0.36
Thiazides/thiazide-like diuretics	41 (13)	36 (16)	5 (7)	0.054
Loop diuretics	154 (51)	98 (43)	56 (76)	<0.0001
Furosemide equivalent dose				0.4
1–50 mg	109 (36)	66 (29)	43 (58)	
51–100 mg	29 (9)	20 (9)	9 (13)	
>100 mg	16 (6)	12 (5)	4 (5)	
SGLT2i	19 (6)	13 (6)	6 (8)	0.44
Insulin	17 (6)	15 (7)	2 (3)	0.23

Values are mean ± standard deviation, *n* (%), or median (25th quartile, 75th quartile). § Micro-albuminuria and macro-albuminuria were defined as uACR ≥30 mg/g and ≥300 mg/g, respectively; ACEi: angiotensin-converting enzyme inhibitor; AFib: Atrial fibrillation; ARB: angiotensin receptor blocker; ARNI: angiotensin receptor neprilysin inhibitor; BMI: body mass index; BSA: body surface area; CAD: coronary artery disease; CRT: cardiac resynchronization therapy; DHP CCB: dihydropyridine calcium channel blocker; eGFR: estimated glomerular filtration rate; ePVS: estimated plasma volume status; FENa: fractional excretion of sodium; HbA1c: glycated haemoglobin (available only in patients with diabetes mellitus); HFpEF: heart failure with preserved ejection fraction; HFrEF: heart failure with reduced ejection fraction; hs-CRP: high sensitivity C-reactive protein; ICD: implantable cardioverter defibrillator; KCCQ: Kansas City Cardiomyopathy Questionnaire; MI: myocardial infarction; MRA: mineralocorticoid receptor antagonist; NT-proBNP: N-terminal prohormone of brain natriuretic peptide; RVF: renal venous flow; SGLT2i: sodium glucose co-transporter 2 inhibitors; uACR: albumin-to-creatinine ratio.

**Table 2:** Ultrasound evaluation according to the presence of continuous *versus* discontinuous RVF.

Variable	Missing	Whole Population (*n* = 304)	Continuous RVF (*n* = 230)	Discontinuous RVF (*n* = 74)	*p*-Value
Left ventricle size and function					
LVMi, g/m^2^	0	121 ± 33	120 ± 30	124 ± 40	0.43
RWT	0	0.39 ± 0.09	0.38 ± 0.09	0.35 ± 0.09	0.017
LV EDVi, mL	0	78 ± 28	76 ± 25	83 ± 35	0.15
LV EF, %	0	61 ± 12	62 ± 12	55 ± 13	<0.0001
LV GLS, %	0	14.1 ± 4.4	14.6 ± 4.2	12.1 ± 4.8	0.005
Stroke volume, mL/beat	0	54 ± 30	54 ± 30	53 ± 29	0.88
Cardiac output, L/min	0	3.8 ± 2.6	3.7 ± 2.6	3.9 ± 2.3	0.60
Mitral E wave, cm/s	0	105 ± 75	96 ± 77	131 ± 61	<0.0001
Average e’, cm/s	0	8.7 ± 2.6	9.1 ± 2.8	8.4 ± 2.6	0.08
Average E/e’	0	10.8 (8.3–15.5)	10.5 (8.1–14.1)	13.5 (9.0–20.0)	<0.0001
Left atrium size and function					
LAVi, mL/m^2^	0	43 ± 16	40 ± 14	51 ± 18	<0.0001
LA reservoir strain, %	0	24 ± 11	26 ± 11	17 ± 8	<0.0001
Right ventricle and pulmonary circulation					
TAPSE, mm	0	20 ± 4	20 ± 4	19 ± 4	0.26
FAC, %	0	50 ± 9	51 ± 8	45 ± 10	<0.0001
RV free wall longitudinal strain, %	9	27 ± 8	28 ± 6	25 ± 6	<0.0001
3D-RV EF, %	9	57 ± 9	59 ± 8	51 ± 9	<0.0001
Systolic PAP, mmHg	4	38 ± 14	34 ± 11	47 ± 16	<0.0001
Diastolic PAP, mmHg	6	11 ± 5	9 ± 4	15 ± 5	<0.0001
Mean PAP, mmHg	6	20 ± 7	18 ± 5	26 ± 9	<0.0001
TAPSE/sPAP, mm/mmHg	0	0.60 ± 0.23	0.64 ± 0.23	0.47 ± 0.20	<0.0001
ePVR, WU	6	1.6 ± 0.9	1.5 ± 0.8	2.0 ± 1.0	0.002
ePAWP, mmHg	6	11.5 ± 5.1	10.0 ± 3.5	16.3 ± 6.3	<0.0001
Congestion assessment	0				
IVC, mm		15 (15–18)	15 (15–18)	20 (17–23)	<0.0001
IVC ≥21 mm		40 (13)	12 (4)	28 (38)	<0.0001
IVC collapse <50%		35 (10)	10 (4)	25 (34)	<0.0001
B-lines		1 (0–5)	1 (0–4)	6 (1–15)	<0.0001

Values are mean ± standard deviation, *n* (%), or median (25th quartile, 75th quartile). EDVi: end-diastolic volume index; ePAWP: echo-derived pulmonary artery wedge pressure; ePVR: echo-derived pulmonary vascular resistance; FAC: fractional area change; GLS: global longitudinal strain; IVC: inferior vena cava; LA: left atrium; LAVi: left atrial volume; LV: left ventricle; EF: ejection fraction; LVMi: left ventricular mass index; PAP: pulmonary artery pressure; RV: right ventricle; RVF: renal venous flow; RWT: relative wall thickness; TAPSE: tricuspid annular plane systolic excursion.

**Table 3:** Laboratory and ultrasound indices of congestion according to individual RVF patterns.

Variable	RVF A (*n* = 230)	RVF B (*n* = 39)	RVF C (*n* = 18)	RVF D (*n* = 17)	*p*-Value
NT-proBNP ^§^, ng/L	332 (160–918)	768 (379–1522) *	1520 (783–2550) *	2147 (1427–4172) *^°	<0.0001
uACR, mg/g	15 (8–53)	22 (8–63)	26 (13–65)	38 (27–121) *	0.03
Serum creatinine, mg/dL	0.93 (0.79–1.18)	0.96 (0.92–1.25)	1.08 (0.93–1.25)	1.14 (0.96–1.39) *	0.03
Average E/e’	10.5 (8.1–14.1)	12.7 (10.1–16.7)	13.1 (8.8–18.6)	15.8 (10.2–21.3) *	0.003
LAVi, mL/m^2^	40 ± 14	49 ± 16 *	50 ± 13 *	60 ± 18 *^	<0.0001
Systolic PAP, mmHg	34 ± 11	44 ± 16 *	49 ± 17 *	54 ± 13 *^	<0.0001
ePVR, WU	1.5 ± 0.8	1.8 ± 0.9	2.1 ± 1.0	2.4 ± 1.1 *	0.016
ePAWP, mmHg	10.0 ± 3.5	14.5 ± 5.9 *	15.7 ± 5.8 *	21.2 ± 5.8 *^°	<0.0001
IVC, mm	15 (15–18)	18 (15–20) *	20 (18–22) *	27 (25–31) *^°	<0.0001
B-lines	1 (0–4)	3 (0–10)	6 (1–15) *	10 (1–18) *	<0.0001
TAPSE/sPAP	0.64 ± 0.23	0.52 ± 0.24 *	0.43 ± 0.17 *	0.36 ± 0.10 *^°	<0.0001

* *p* < 0.05 vs RVF A; ^ *p* < 0.05 vs RVF B; ° *p* < 0.05 vs RVF C. ^§^ Log-transformed. Analyses are adjusted for hypertension, diabetes mellitus, definite diagnosis of HF (i.e., HF Stage C) and serum creatinine. ePAWP: echo-derived pulmonary artery wedge pressure; ePVR: echo-derived pulmonary vascular resistance; HF: heart failure; IVC: inferior vena cava; LAVi: left atrial volume; NT-proBNP: N-terminal prohormone of brain natriuretic peptide; PAP: pulmonary artery pressure; RVF: renal venous flow; uACR: urinary albumin-to-creatinine ratio.

## Data Availability

The data that support the findings of this study are available from the corresponding author, upon reasonable reques.

## Supplementary Materials

The following supporting information can be downloaded at: https://www.mdpi.com/article/10.3390/medicina59101704/s1, Table S1: Laboratory and ultrasound indices of congestion according to HF and individual RVF patterns. Table S2: Laboratory and ultrasound indices of congestion according to individual RVF patterns in patients with HF Stage C (*n* = 241). Table S3: Laboratory and ultrasound indices of congestion according to individual RVF patterns in patients with atrial fibrillation (*n* = 50). References [14,15,46,47,48] are cited in the supplementary materials.
