# Supplementary material for: Bio-Humoral and Non-Invasive Haemodynamic Correlates of Renal Venous Flow Patterns across the Heart Failure Spectrum
Source: Medicina (Kaunas). 2023 Sep 24;59(10):1704. doi: 10.3390/medicina59101704 (PMC10608031; doi:10.3390/medicina59101704)
Supplement: Supplementary file 1 [file medicina-59-01704-s001.zip › medicina-2598246-supplementary.pdf]

Supplementary Materials: Bio-Humoral and Non-Invasive Haemodynamic Correlates of Renal Venous Flow Patterns Across the Heart Failure Spectrum

Baseline echocardiography protocol. All patients underwent a comprehensive transthoracic echocardiography examination (Hitachi Medical Systems LISENDO 880, Tokyo, Japan) according to the international recommendations.[1] Stroke volume was calculated by multiplying the left ventricular (LV) outflow tract area by the LV outflow tract velocity–time integral measured by pulsed–wave Doppler. Cardiac output was calculated by multiplying stroke volume by heart rate. With the patient in the supine position, the maximum inferior vena cava (IVC) diameter during the respiratory cycle was measured between 1 and 3 cm before the merger with the right atrium. The IVC collapse was visually estimated as ≥50 or <50% following deep inspiration (a brief sniff). IVC diameter and its variations were used to estimate right atrial pressure (RAP), as recommended[1]. Systolic pulmonary artery pressure (sPAP) was measured from the peak tricuspid regurgitation velocity (TRV) with the simplified Bernoulli equation, adding the estimated RAP. Diastolic pulmonary artery pressure (dPAP) was calculated by adding RAP to the pulmonary regurgitation end–diastolic gradient (PREDG). Then mean pulmonary artery pressure (mPAP) was calculated as (sPAP + 2 · dPAP)⁄3. Left atrial volume index (LAVi) was estimated with the disc summation algorithm (Simpson's technique) in a biplane approach from the apical four–chamber and two–chamber view[1]. To maximise image quality and decrease the likelihood of discarding patients for poor acoustic windows, we also employed off–axis approaches, such as the right ventricle inflow tract for TRV, subcostal view for PREDG, and left lateral approach for IVC dimension and variations. We non–invasively estimated echo–derived pulmonary artery wedge pressure (ePAWP) using a previously validated equation, which includes the following variables: tricuspid regurgitation velocity (TRV), LVEF, right ventricle fractional area change, left atrial volume index (LAVi), E/e', inferior vena cava and mPAP[2]. Then, echo–derived pulmonary vascular resistance (ePVR) was calculated as (mPAP – ePAWP)/CO[2]. Valvular regurgitation was qualitatively assessed using color–Doppler, and whenever regurgitation was more than mild, it was quantified using the width of the vena contracta and the effective regurgitant orifice area[3]. Valvular stenosis was assessed using continuous–wave Doppler and quantified using peak transvalvular velocity and mean transvalvular pressure gradient[4]. All measurements were reported as the average of three beats for patients in normal sinus rhythm and five beats for patients with atrial fibrillation.

3D Transthoracic Echocardiography (3DTE). 3D full–volume data sets were acquired using a single–crystal matrix–array transducer. The acquisitions were obtained in full–volume mode from the 4–chamber apical view. Care was taken to include the entire LV and RV cavity within the pyramidal scan volume. To ensure a relatively high–volume rate, data sets throughout one cardiac cycle were acquired using two wedge–shaped subvolumes, acquired with electrocardiographic gating during a single 5– to 7–second breath–hold and over at least five cardiac cycles. The data were analysed offline using a vendor–independent software (TomTec Imaging Systems, Unterschleissheim, Germany). To determine the RV parameters, we used the 4D RV analysis software (TomTec Imaging Systems, Unterschleissheim, Germany). Non–foreshortened apical 4– and 2–chamber views at the end–diastole were identified to select the LV apex and the centre of the mitral annular line, placing the largest LV long–axis dimensions. In the apical 3–chamber view, both the anterior and the posterior aortic annuli were identified. In the RV apical 4–chamber and coronal views, the point of the RV apex and the center of the tricuspid annular line were identified. In the short–axis view, both the anterior and the posterior junction between the RV free wall and interventricular septum were identified. Then, the distance between the interventricular septal and RV free wall was delineated perpendicular to the midpoint of the interventricular septum. The software automatically reconstructed the RV endocardial surface at end–diastole, and manual editing was performed when required. The endocardial surface was manually readjusted as necessary when tracking was deemed inadequate. RV volumes were computed throughout the cardiac cycle, from which the 3D RV end–diastolic volume, end–systolic volume and RVEF were automatically calculated. The same software performed STE analysis throughout the entire cardiac cycle and determined the RV free wall longitudinal strain. The whole post–processing quantification required 4±2 minutes.

Speckle tracking echocardiography (STE). We measured LV global longitudinal strain (GLS) from the apical long–axis view and two– and four–chamber views, ensuring a frame rate >50 Hz (2D strain analysis, TomTec Imaging Systems, Unterschleissheim, Germany). We reported the average LVGLS values from the three apical views at rest. We excluded poorly tracked segments, and patients were not analysed if more than one segment per view was deemed unacceptable. We measured left atrial (LA) reservoir strain using the same software as the average of six segments in the four–chamber and two–chamber views, ensuring a frame rate >50 Hz[5]. LA strain was measured using the QRS as the fiducial point. STE–derived measurements were reported as the average of three beats for patients in normal sinus rhythm

and five beats for patients with atrial fibrillation. All measurements were performed offline by expert readers blinded to clinical and other instrumental data.

Table S1. Laboratory and ultrasound indices of congestion according to HF and individual RVF patterns.

**Variable**	HF Stages A/B<br>**(n=63)**	HF Stage C + cRVF<br>**(n=171)**	HF Stage C + dRVF<br>**(n=70)**	**p–value**
NT–proBNP[§], ng/L	105 (48–157)	669 (330–1384)*	1247 (759–2279)*°	**<0.0001**
Creatinine, mg/dL	0.89 (0–82–1.04)	0.99 (0.81–1.27)	1.12 (0.91–1.32)	0.25
Average E/e′	8.3 (6.9–10.0)	11.4 (9.2–16.0)	15.2 (10.0–20.3)*	**0.01**
LAVi, mL/m^2	35±12	42±14*	52±17*°	**<0.0001**
Systolic PAP, mmHg	31±11	36±11	49±16*°	**<0.0001**
ePVR, WU	1.3±0.9	1.6±0.7	2.0±1.0*°	**<0.0001**
ePAWP, mmHg	9±3	11±3	17±6*°	**<0.0001**
IVC, mm	15 (15–18)	15 (15–18)	20 (15–22)*°	**<0.0001**
B–lines	1 (0–3)	1 (0–4)	6 (0–15)*°	**<0.0001**
TAPSE/sPAP, mm/mmHg	0.76±0.28	0.60±0.21*	0.44±0.20*°	**<0.0001**

* p <0.05 vs no HF/cRVF; ° p <0.05 vs HF/cRVF.
Adjusted for hypertension, diabetes mellitus, and serum creatinine.
§ Log–transformed
ePAWP: echo–derived pulmonary artery wedge pressure; ePVR: echo–derived pulmonary vascular resistance; HF: heart failure; IVC: inferior vena cava; LAVi: left atrial volume; NT–proBNP: N–terminal prohormone of brain natriuretic peptide; PAP: pulmonary artery pressure; RVF: renal venous flow; uACR: urinary albumin–to–creatinine ratio.

Table S2. Laboratory and ultrasound indices of congestion according to individual RVF patterns in patients with HF Stage C (n=241).

Variable	RVF A (n=171)	RVF B (n=35)	RVF C (n=18)	RVF D (n=17)	p–value
NT–proBNP[§], ng/L	663 (303–1641)	1043 (464–1547)	1520 (783–2550)*	2147 (1427–4172)*^°	**<0.0001**
uACR[#], mg/g	21 (11–58)	24 (13–64)	26 (9–65)	38 (27–121)	0.07
Serum creatinine, mg/dL	0.94 (0.81–1.21)	0.96 (0.83–1.24)	1.08 (0.92–1.25)	1.14 (0.94–1.39)*	**0.04**
Average E/e′	11.3 (8.8–15.4)	11.9 (9.6–14.1)	13.1 (8.8–18.6)	15.8 (10.2–21.3)*	**0.003**
LAVi, mL/m^2	42±14	49±15	50±13*	60±18*^	**<0.0001**
Systolic PAP, mmHg	36±11	45±16*	49±17*	54±13*^	**<0.0001**
ePVR, WU	1.5±0.7	1.8±0.9	2.1±1.0	2.4±1.1*	**<0.0001**
ePAWP, mmHg	11±4	15±6*	15.7±5.8*	21.2±5.8*^°	**<0.0001**
IVC, mm	15 (15–18)	18 (15–20)*	20 (18–22)*	27 (25–31)*^°	**<0.0001**
B–lines	2 (0–5)	5 (0–11)*	6 (1–15)*	10 (1–18)*	**<0.0001**
TAPSE/sPAP, mm/mmHg	0.59±0.21	0.48±0.24*	0.43±0.17*	0.36±0.10*^°	**<0.0001**

* p <0.05 vs RVF A; ^ p <0.05 vs RVF B; ° p <0.05 vs RVF C.
§ Log–transformed
Analyses are adjusted for hypertension, diabetes mellitus, LV EF and serum creatinine.
ePAWP: echo–derived pulmonary artery wedge pressure; ePVR: echo–derived pulmonary vascular resistance; HF: heart failure; IVC: inferior vena cava; LAVi: left atrial volume; LV EF: left ventricular ejection fraction; NT–proBNP: N–terminal prohormone of brain natriuretic peptide; PAP: pulmonary artery pressure; RVF: renal venous flow; uACR: urinary albumin–to–creatinine ratio.

Table S3. Laboratory and ultrasound indices of congestion according to individual RVF patterns in patients with atrial fibrillation (n=50)

**Variable**	**RVF A** (n=23)	**RVF B** (n=8)	**RVF C** (n=8)	**RVF D** (n=11)	**p–value**
NT–proBNP§, ng/L	1017 (666–1251)	1577 (750–2606)	1689 (1305–2701)	2344 (1553–4666)*	**0.001**
uACR#, mg/g	19 (8–59)	22 (12–64)	25 (11–65)	35 (25–122)	0.08
Serum creatinine, mg/dL	0.93 (0.83–1.21)	1.02 (0.90–1.37)	1.07 (0.94–1.24)	1.13 (1–04–1.30)	0.13
Average E/e′	11.3 (9.4–15.6)	10.6 (9.6–13.7)	11.7 (8.3–14.0)	16.4 (11.6–20.0)*	**0.01**
LAVi, mL/m^2	51±17	54±13	58±18	64±25*	**0.01**
Systolic PAP, mmHg	41±11	45±12	52±17	57±12*	**0.03**
ePVR, WU	1.8±0.8	2.1±0.8	2.4±1.2	2.4±1.2	0.18
ePAWP, mmHg	14±4	16±5	18±5	20±5*	**0.004**
IVC, mm	18 (15–19)	21 (19–22)	21 (20–22)	28 (25–32)*^°	**<0.0001**
B–lines	3 (0–7)	11 (7–15)	3 (2–7)	11 (4–19)	0.11
TAPSE/sPAP, mmHg	0.44±0.15	0.42±0.21	0.41±0.17	0.35±0.12*	**0.01**

* p <0.05 vs RVF A; ^ p <0.05 vs RVF B; ° p <0.05 vs RVF C.
§ Log–transformed
Analyses are adjusted for hypertension, diabetes mellitus, definite diagnosis of HF (i.e., HF Stage C) and serum creatinine.
AFib: atrial fibrillation; ePAWP: echo–derived pulmonary artery wedge pressure; ePVR: echo–derived pulmonary vascular resistance; HF: heart failure; IVC: inferior vena cava; LAVi: left atrial volume; NT–proBNP: N–terminal prohormone of brain natriuretic peptide; PAP: pulmonary artery pressure; RVF: renal venous flow; uACR: urinary albumin–to–creatinine ratio.

REFERENCES

1. Lang RM, Badano LP, Mor–Avi V, Afilalo J, Armstrong A, Ernande L, Flachskampf FA, Foster E, Goldstein SA, Kuznetsova T, Lancellotti P, Muraru D, Picard MH, Rietzschel ER, Rudski L, Spencer KT, Tsang W, Voigt JU. Recommendations for cardiac chamber quantification by echocardiography in adults: An update from the American society of echocardiography and the European association of cardiovascular imaging. *Eur Heart J Cardiovasc Imaging* 2015;**16**:233–271.

2. Chubuchny V, Pugliese NR, Taddei C, Poggianti E, Spini V, Barison A, Formichi B, Airò E, Bauleo C, Prediletto R, Pastormerlo LE, Coceani M, Ciardetti M, Petersen C, Pasanisi E, Lombardi C, Emdin M, Giannoni A. A novel echocardiographic method for estimation of pulmonary artery wedge pressure and pulmonary vascular resistance. *ESC Hear Fail* ESC Heart Fail; 2021;**8**:1216–1229.

3. Lancellotti P, Pibarot P, Chambers J, Canna G La, Pepi M, Dulgheru R, Dweck M, Delgado V, Garbi M, Vannan MA, Montaigne D, Badano L, Maurovich–Horvat P, Pontone G, Vahanian A, Donal E, Cosyns B. Multi–modality imaging assessment of native valvular regurgitation: an EACVI and ESC council of valvular heart disease position paper. *Eur Hear J – Cardiovasc Imaging* Oxford University Press (OUP); 2022;

4. Baumgartner H, Falk V, Bax JJ, Bonis M De, Hamm C, Holm PJ, Iung B, Lancellotti P, Lansac E, Muñoz DR, Rosenhek R, Sjögren J, Tornos Mas P, Vahanian A, Walther T, Wendler O, Windecker S, Zamorano JL, Roffi M, Alfieri O, Agewall S, Ahlsson A, Barbato E, Bueno H, Collet JP, Coman IM, Czerny M, Delgado V, Fitzsimons D, Folliguet T, et al. 2017 ESC/EACTS Guidelines for the management of valvular heart disease. Eur. Heart J. 2017.

5. Reddy YNV, Obokata M, Egbe A, Yang JH, Pislaru S, Lin G, Carter R, Borlaug BA. Left atrial strain and compliance in the diagnostic evaluation of heart failure with preserved ejection fraction. *Eur J Heart Fail* John Wiley and Sons Ltd; 2019;**21**:891–900.
